# Prognostic and diagnostic value of circulating IGFBP2 in pancreatic cancer

**DOI:** 10.1515/med-2023-0893

**Published:** 2024-08-30

**Authors:** Jie Xu, Yuning Song, Bodong Zhou, Shuai Yuan, Song Gao

**Affiliations:** Senior Ward, Tianjin Medical University Cancer Institute and Hospital, National Clinical Research Center for Cancer, Key Laboratory of Cancer Prevention and Therapy, Tianjin, 300060, China; Department of Pancreatic Cancer, Tianjin Medical University Cancer Institute and Hospital, National Clinical Research Center for Cancer, Key Laboratory of Cancer Prevention and Therapy, Tianjin, 300060, China

**Keywords:** IGFBP2, pancreatic adenocarcinoma, prognosis, survival, biomarker, chronic pancreatitis

## Abstract

Insulin-like growth factor binding protein 2 (IGFBP2) is overexpressed in tumor tissues of several malignancies, including pancreatic cancer. Because of its role in tumor progression, IGFBP2 has been investigated as a tumor biomarker. However, little is known about its utility in pancreatic cancer. Plasma IGFBP2 levels were determined using enzyme-linked immunosorbent assay in 75 patients with pancreatic ductal adenocarcinoma (PDAC), 73 matched healthy controls, and 17 chronic pancreatitis patients. Our results showed that the plasma IGFPB2 level was significantly higher in PDAC patients than in patients with chronic pancreatitis and healthy controls. At a cut-off value of 333.9 ng/mL, the specificity and sensitivity were 78.08 and 65.33%, respectively. IGFBP2 level alone did not outperform carbohydrate antigen 19-9 (CA19-9) in diagnostic accuracy, but it successfully identified 9 out of 24 PDAC patients who were misidentified by CA19-9. The combination of IGFBP2 and CA19-9 was more accurate in the detection of PDAC than CA19-9 alone. IGFBP2 was more accurate than the other in discriminating between chronic pancreatitis and PDAC. Plasma IGFBP2, rather than CA19-9, was higher in the new-onset diabetes, lymph node involvement, and distant metastasis subgroups. IGFBP2 level was notably higher in stage IV cases than in stage I/II or stage III disease. However, CA19-9 did not show a difference between stages. After adjusting for lymph node involvement and distant metastasis, plasma IGFBP2 was identified as an independent prognostic marker for PDAC. The median survival time for patients with an IGFBP2 level ≥333.9 ng/mL was significantly shorter than that for patients with an IGFBP2 level <333.9 ng/mL. Marked elevation of plasma IGFBP2 in PDAC is associated with poorer survival. IGFBP2 may be considered as a supplementary biomarker for the diagnosis and prognostic prediction in Chinese pancreatic cancer patients.

## 1 Introduction

Pancreatic ductal adenocarcinoma (PDAC) is a devastating malignancy worldwide. The incidence is increasing annually, while the 5-year overall survival rate remains less than 10% [1]. Despite developments in surgical techniques and adjuvant chemotherapy, pancreatic cancer has shown a slow improvement in survival rates [2]. The main reason for poor survival is late diagnosis.

Carbohydrate antigen 19-9 (CA19-9) is commonly used as a conventional biomarker for PDAC, but its universal application is limited for several reasons, including false-negative results in 10% of cases with the Lewis-negative genotype as well as false-positive results in obstructive jaundice, chronic pancreatitis, and other malignancies. Although risk factors for PDAC such as diabetes mellitus and smoking, have been claimed, doctors are reluctant to use CA19-9 for screening the risk category because it is less effective [3].

The insulin-like growth factor-binding proteins (IGFBPs) are a family of proteins that bind to and serve as carriers of IGFs, prolonging the IGF half-life in the circulation and modulating local IGF concentrations and activities [4]. IGFBP2 is the second most abundant circulating IGFBPs. IGFBP2 has shown diagnostic and prognostic potential in glioma [5], prostate cancer, and ovarian cancer [6]. IGFBP2 was first discovered in the proteomic studies to be elevated of pancreatic juice from PDAC patients [7]. Later, it was found to be elevated in PDAC tumor samples, but not in chronic pancreatitis tissue [8]. In a randomized phase 2 trial, the plasma IGFBP2 level was found to be correlated with the response to treatment with gemcitabine and the IGF1 receptor inhibitor ganitumab in metastatic pancreatic cancer [9].

Kendrick et al. first discussed using plasma IGFBP2 and methoselin as a panel of potential diagnostic biomarkers in a US cohort of PDAC patients [10]. Nevertheless, it is still not clear if plasma IGFBP2 level correlates with primary clinical variables and survival. Our purpose was to investigate plasma IGFBP2 levels in PDAC patients, chronic pancreatitis patients, and matched healthy controls in a Chinese cohort. Besides validating that plasma IGFBP2 level was significantly elevated in PDAC patients, we also showed important correlations between plasma IGFBP2 and certain clinical parameters. Finally, we showed that high levels of plasma IGFBP2 was correlated with poor survival in Chinese PDAC patients.

## 2 Materials and methods

### 2.1 Study subjects

A total of 75 patients with newly diagnosed PDAC between 2009 and 2012 at Tianjin Cancer Hospital, Tianjin, China, were randomly selected into this retrospective study as the PDAC patient group. PDAC was confirmed in these patients through histological diagnosis on fine-needle biopsy specimens or immunohistochemical testing on surgical samples. We also included 17 patients with chronic pancreatitis (ChPT) who were admitted to the outpatient pancreatic cancer clinic of Tianjin Cancer Hospital for evaluation of a “pancreatic mass” identified at another institution. Another 73 persons without PDAC or ChPT were selected as age- and sex-matched healthy controls from among the individuals who visited Tianjin Cancer Hospital for cancer screening. Exclusion criteria included patients with other malignancies diagnosed or treated within the last 5 years. The study was approved by the research ethics committees of Tianjin Cancer Hospital, and written informed consent was obtained from each patient.

### 2.2 Blood sample collection and processing

Peripheral blood samples were collected with either heparin or ethylenediaminetetraacetic acid (EDTA) as an anti-coagulation agent. Plasma was isolated by subjecting the samples to centrifugation at 1,500*g* for 5 min at room temperature. A pilot study on three healthy controls showed no significant difference in IGFBP2 levels in plasma specimens obtained with either heparin or EDTA from the same person. Plasma samples were frozen and maintained at −80°C until the assays were conducted.

### 2.3 Enzyme-linked immunosorbent assay (ELISA) for IGFBP2

Plasma IGFBP2 was measured by an ELISA kit (R&D Systems, Minneapolis, USA), following the manufacturer’s instructions.

### 2.4 Statistical analysis

Data were analyzed using IBM SPSS version 22 (SPSS Inc., Chicago, IL, USA) for Windows and GraphPad Prism version 6.0 (GraphPad Software, Inc., La Jolla, CA, USA). Values of IGFBP2 were expressed as mean ± standard deviation (SD). Demographic data were analyzed by the chi-square test and continuous data were compared with an independent-sample Student’s *t*-test. We compared the differences in plasma levels of IGFBP2 between cases and controls using the nonparametric Mann–Whitney *U* test and evaluated the relationship between IGFBP2 levels and clinical parameters through univariate linear regression analysis. We constructed survival curves according to plasma levels of IGFBP2 and CA19-9 using the Kaplan–Meier method and compared the median survival times by the log-rank test. Hazard ratios (HRs) and 95% confidence intervals (CIs) for survival were estimated by multivariate Cox proportional hazard regression analysis, adjusting for lymph node involvement and distant metastasis. All *P* values were two-tailed, with 0.05 specified as the threshold for statistical significance.

## 3 Results

### 3.1 Clinicopathological characteristics of patients

Clinicopathologic characteristics of the 75 patients with newly diagnosed, pathologically confirmed PDAC in our cohort are summarized in Table 1. Out of the 40 cases of type II diabetes mellitus, 25 were new-onset diabetes (i.e., <24 months in duration) at study entry. The small number of patients with stage I disease prompted us to combine stage I and II for the data analysis. Of the 35 patients who underwent radical resection, 21 patients underwent pancreaticojejunostomy and the other 14 distal pancreatectomy and splenectomy. Of the other 40 patients, 12 underwent palliative gastrojejunostomy or choledochojejunostomy, or both, to relieve alimentary or bile tract obstruction and 28 patients had no operation. The mean follow-up time was 9 months. The survival data were available for all 75 patients; 8 (10.6%) were still alive at the time of data analysis. The healthy controls were matched with the PDAC cases by age, sex, and race. Plasma CA19-9 data were obtained from the clinical records for each patient and the health controls.

**Table 1:** Basic clinicopathological information of patients

Variable	No.
Male/female	49/26
Median age at diagnosis, years (range)	60 (39–80)
Diabetes mellitus (new onset/long term/no)	15/25/35
Tumor location* (H&N/B&T)	48/27
Tumor size, cm (mean ± SD)	4.09 ± 0.19
TNM classification T stage (T1/T2/T3/T4)	0/4/31/40
TNM classification N stage (N0/N1)	33/42
TNM classification M stage (M0/M1)	54/21
UICC stage (IB/IIA/IIB/III/IV)	3/17/11/23/21
Resected/unresected	35/40

*Tumor location: H, pancreatic head; N, pancreatic neck; B, pancreatic body; T, pancreatic tail.

### 3.2 Plasma IGFBP2 levels in patients with pancreatic cancer, chronic pancreatitis, and healthy controls

**Figure 1:**
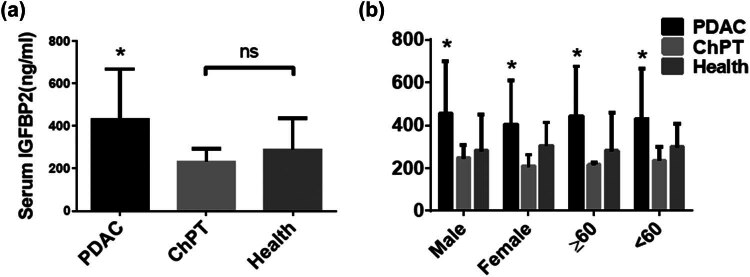
Plasma IGFBP2 levels in PDAC patients (*N* = 75), chronic pancreatitis patients (*N* = 17), and healthy controls (*N* = 73). (a) The column bar graphs and error bars show mean ± SD. Serum IGFBP2 level was significantly elevated in PDAC. (b) Comparison of PDAC and ChPT patients and healthy controls on the basis of sex and age.

To assess the differences in IGFBP2 levels among patients with PDAC or ChPT and healthy controls, plasma IGFBP2 levels were determined blindly by ELISA. The mean plasma concentration of IGFBP2 in PDAC patients (435.7 ± 234.2 ng/mL) was significantly higher than that of either ChPT patients (233.0 ± 59.80 ng/mL, *P* = 0.0007) or healthy controls (289.9 ± 148.2 ng/mL, *P* < 0.0001; Figure 1a). However, no difference was observed between ChPT patients and healthy controls (*P* = 0.1254). In an analysis of these data by sex and age, IGFBP2 level was still higher in patients with PDAC than those with ChPT or healthy controls in each group (all *P* < 0.001; Figure 1b). Moreover, no obvious difference was found between healthy controls and nine ChPT patients in different sex or age groups. These results demonstrate that circulating IGFBP2 values were significantly higher in patients with PDAC, regardless of age or sex.

### 3.3 Specificity and sensitivity of plasma IGFBP2 as a diagnostic biomarker for pancreatic cancer

**Figure 2:**
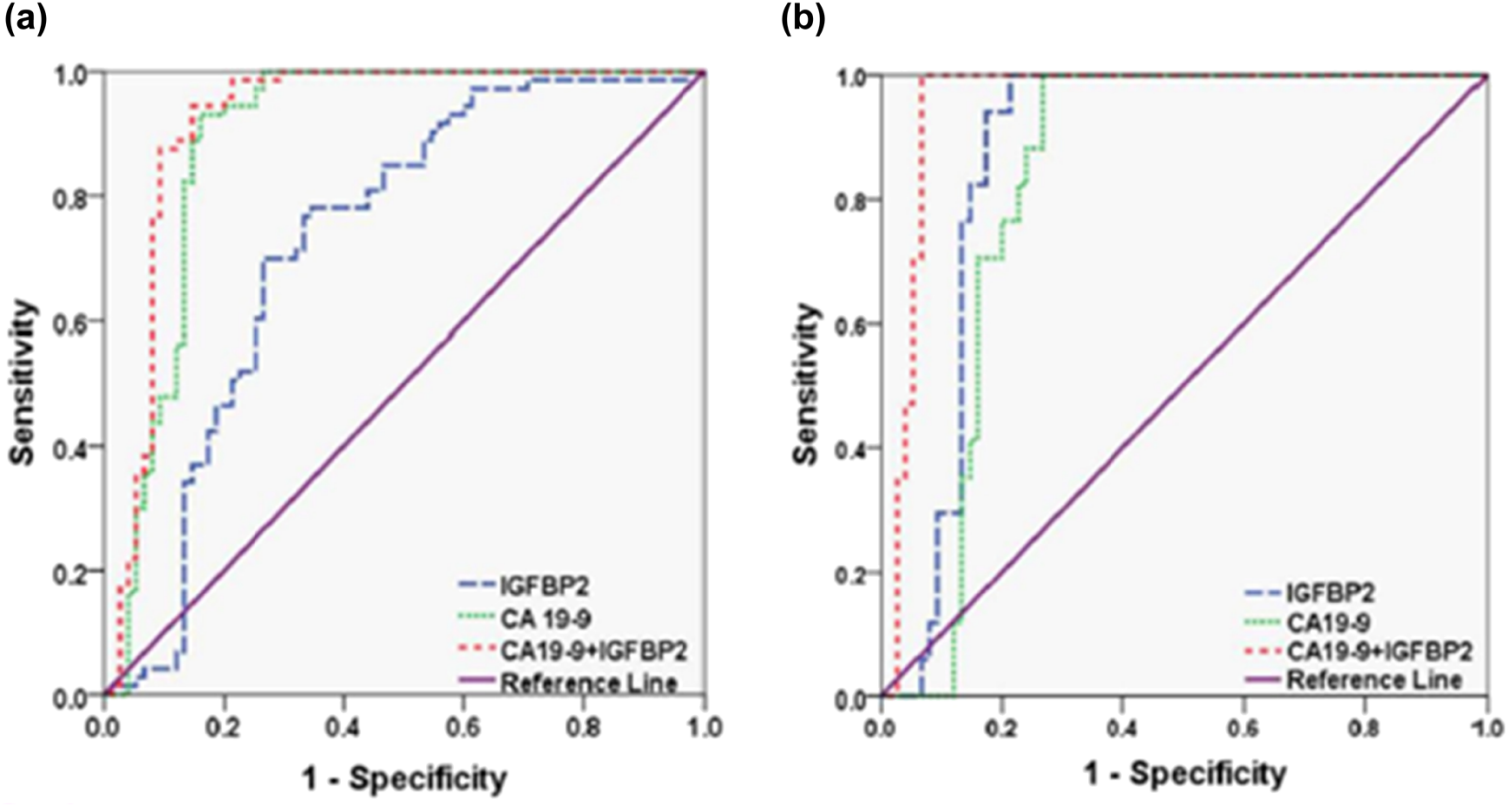
ROC operator characteristic curves for diagnosis of PDAC versus healthy controls and chronic pancreatitis. (a) ROC curves comparing PDAC patients to healthy controls for IGFBP2. (b) ROC curves comparing PDAC patients to ChPT patients for IGFBP2 and the combination of IGFBP2 plus CA19-9. AUC: area under the curve.

From the receiver operating characteristic (ROC) curve, we determined the specificity and sensitivity of IGFBP2 for diagnosis. At a cut-off value of 333.9 ng/mL, the specificity and sensitivity of IGFBP2 were 78.08 and 65.33%, respectively. The area under the ROC curve (AUC) of IGFBP2 was 0.727 (Figure 2a). The 95% specificity threshold of IGFBP2 yielded a sensitivity of 38.6%. CA 19-9, the most widely used biomarker for PDAC, yielded an AUC of 0.893 in our cohort. At the widely used clinical threshold of 37 U/mL, CA 19-9 produced a sensitivity of 73% and a specificity of 97% in this cohort. The 95% specificity threshold of CA 19-9 yielded a sensitivity of 75%. Although the diagnostic power of IGFBP2 alone was not as great as that of CA 19-9, IGFBP2 at its 95% specificity concentration (494 ng/mL) correctly identified 9 out of the 24 cases misidentified by CA 19-9 at the 37 U/mL threshold. Furthermore, the AUC of the combination of IGFBP2 and CA19-9 reached 0.921. ROC curves were generated to compare the utility of plasma IGFBP2 levels in differentiating PDAC from ChPT (Figure 2b). IGFBP2 outperformed CA19-9 in diagnostic accuracy, with AUCs of 0.817 and 0.779, respectively (*P* < 0.001). The combination of IGFBP2 and CA19-9 yielded an AUC of 0.918 in differentiating PDAC from ChPT.

### 3.4 Plasma IGFBP2 in subgroups of pancreatic cancer patients

**Figure 3:**
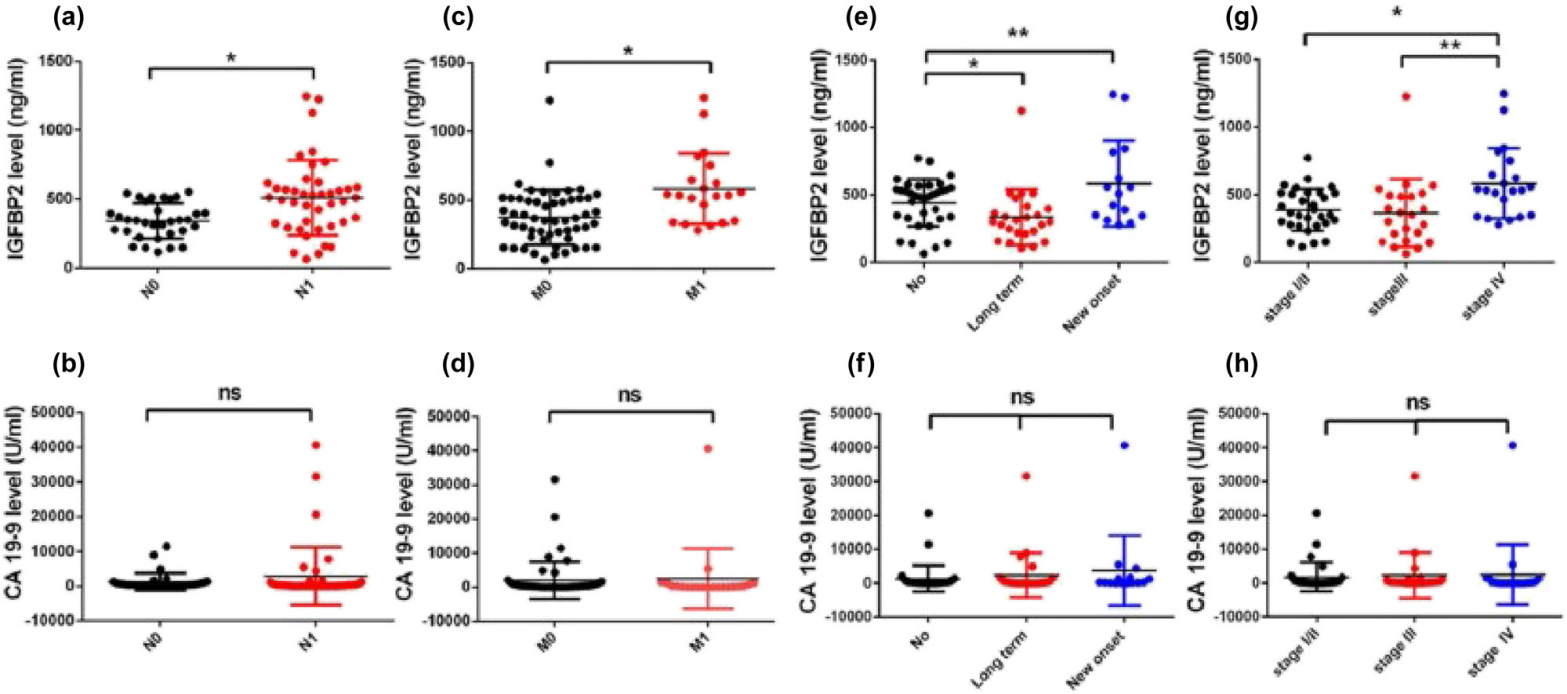
Scatter plots for plasma IGFBP2 and CA19-9 levels in subgroups of PDAC patients. (a) IGFBP2 levels and (b) CA 19-9 levels in patients with and without lymph node involvement. (c) IGFBP2 levels and (d) CA 19-9 levels in patients with and without distant metastasis. (e) IGFBP2 levels and (f) CA 19-9 levels in patients with different diabetes status. (g) IGFBP2 levels and (h) CA 19-9 levels in patients at different tumor stages.

Plasma IGFBP2 levels in various PDAC patient subgroups were analyzed to determine the correlation between IGFBP2 and clinical parameters (Table 2; Figure 3). No difference was identified in the sex, age, or tumor size subgroups. Patients with long-term diabetes had a relatively lower IGFBP2 than those without diabetes (336.4 ± 205.3 ng/mL vs 442.6 ± 176.2, *P* = 0.0359), while patients with new-onset diabetes exhibited a significantly higher IGFBP2 (585.4 ± 317.2 ng/mL) than patients without diabetes (*P* = 0.0466). CA19-9 levels did not differ significantly between the diabetes subgroups (Table 2; Figure 3e). IGFBP2 was elevated in patients with lymph node involvement (509.1 ± 271.3 ng/mL) and in those with distant metastasis (583.4 ± 258.1 ng/mL) compared to their respective control groups. However, CA19-9 does not show any difference between these groups (Figure 3f and g). Patients with stage I/II PDAC (388.3 ± 155.7 ng/mL, *P* = 0.0013) and those with stage III PDAC (364.9 ± 248.4 ng/mL, *P* = 0.0066) showed significantly lower IGFBP2 values than patients with stage IV PDAC (583.4 ± 258.1 ng/mL). This suggests that IGFBP2 levels increase as the disease progresses to an advanced stage. CA19-9 levels did not differ according to disease stage (Figure 3h).

**Table 2:** Pancreatic tumor patient characteristics and plasma IGFBP2 levels (mean ± SD)

Variable	Patient (N)	IGFBP2 (ng/mL)	*P* value	CA 19-9 (U/mL)	*P* value
**Patient sex**					
Male	49	453.0 ± 247.8		2,675 ± 1,106	
Female	26	403.2 ± 206.9	0.3852	820.1 ± 439.9	0.2369
**Age (years)**					
<60	34	429.7 ± 236.1		1,896 ± 1,210	
≥60	41	440.7 ± 235.4	0.8423	2,362 ± 941.6	0.7586
**Diabetes mellitus**					
No	35	442.6 ± 176.2		1,281 ± 655.7	
Long term	25	336.4 ± 205.3	0.0359*	2,417 ± 1,308	0.4031
New onset	15	585.4 ± 317.2	0.0466**	3,736 ± 2,671	0.2249
**Distant metastasis**					
No	54	374.2 ± 199.1		2,016 ± 733.5	
Yes	21	583.4 ± 258.1	0.003	2,497 ± 1,926	0.7748
**Lymph node involvement**					
No	33	342.3 ± 128.3		1,215 ± 436.9	
Yes	42	509.1 ± 271.3	0.0017	2,886 ± 1,286	0.2700
**Tumor size (cm)**					
<4.09	35	426.7 ± 206.5		1,880 ± 1,083	
≥4.09	40	443.6 ± 258.4	0.7570	2,375 ± 1,042	0.7742
**Stage**					
I/II	31	388.3 ± 155.7	0.0013***	1,795 ± 771.4	0.7039
III	23	364.9 ± 248.4	0.0066****	2,314 ± 1,394	0.9382
IV	21	583.4 ± 258.1		2,497 ± 1,926	

*and**, *P* value for long-term diabetes or new onset diabetes versus no diabetes; *** and ****, *P* value for stage I/II versus stage IV and stage III versus stage IV.

### 3.5 Correlation between plasma IGFBP2 levels and survival of patients with pancreatic cancer

**Figure 4:**
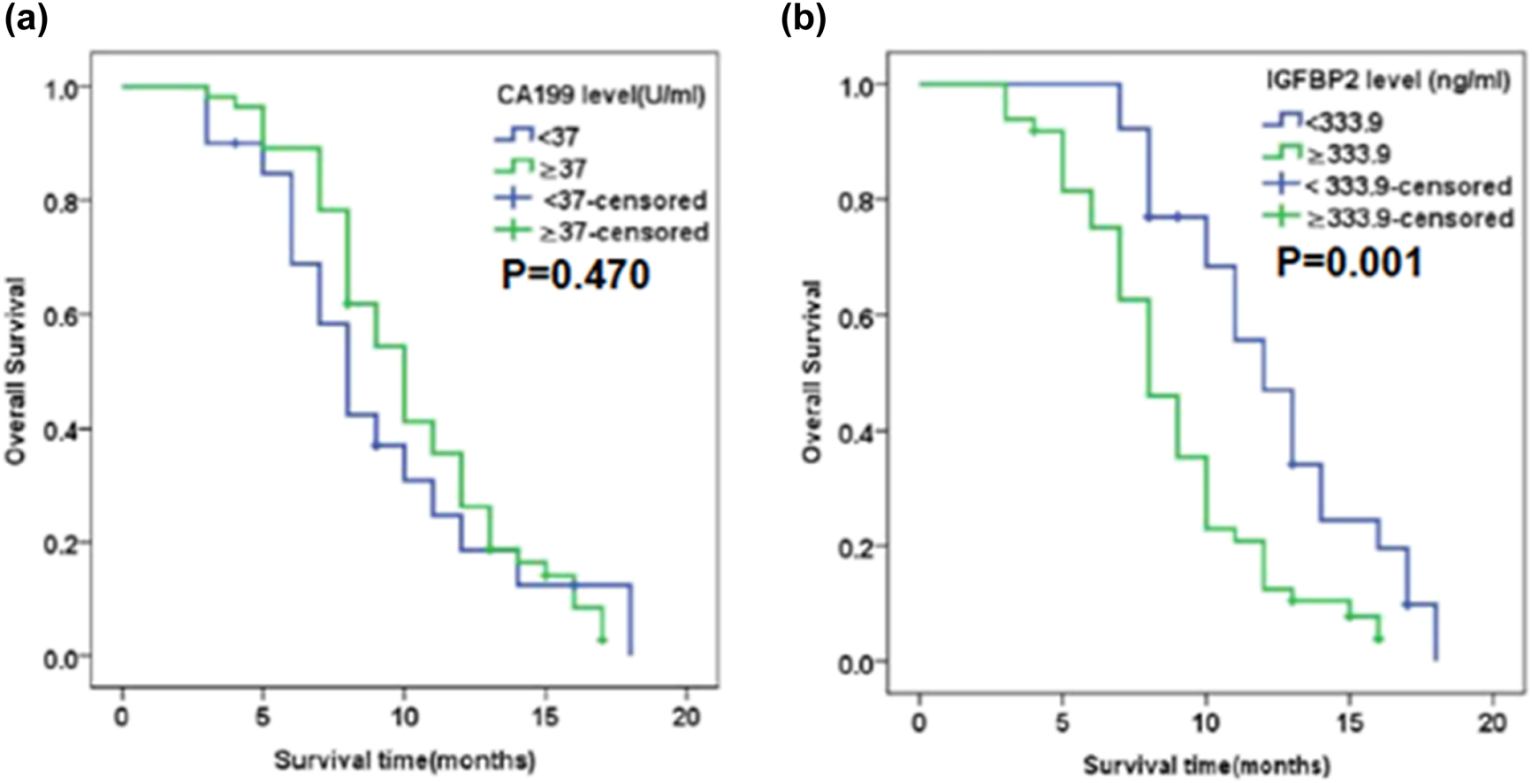
Association of plasma CA19-9 and IGFBP2 levels with survival time. Log Rank test was performed for curves comparison.The Kaplan–Meier survival curves for (a) PDAC patients grouped by the plasma CA19-9 cutoff value of 37 U/mL and (b) patients grouped by the plasma IGFBP2 cutoff value of 333.9 ng/mL.

The relationship between elevated plasma IGFBP2 and patient prognosis was determined through survival analysis. Univariate analysis indicated that survival was significantly correlated with lymph node involvement (HR = 2.099, 95% CI 1.269–3.473, *P* = 0.004), distant metastasis (HR = 4.530, 95% CI 2.581–7.949, *P* < 0.001), and plasma IGFBP2 level (HR = 2.318, 95% CI 1.344–3.998, *P* = 0.003). In multivariate analysis, a plasma IGFBP2 level greater than the cut-off value of 333.9 ng/mL was found to be an independent predictor of survival (HR = 2.353, 95% CI 1.344–4.117, *P* = 0.003) after adjusting for lymph node involvement and distant metastasis. Distant metastasis was identified as another independent factor. The results of the univariate and multivariate analyses are summarized in Table 3. The median survival time for patients with an IGFBP2 level ≥333.9 ng/mL was significantly shorter than that for patients with an IGFBP2 level <333.9 ng/mL (8 months vs 11 months, *P* < 0.001; Figure 4). No significant median survival was shown between patients grouped by CA19-9 cutoff value at 37 U/mL (*P* = 0.470).

**Table 3:** Univariate and multivariate analyses for prognostic factors in PDAC cases (*N* = 75)*

			Univariate			Multivariate	
Variable	No.	Hazard ratio	95% CI	*P* value	Hazard ratio	95% CI	*P* value
**Sex**							
Female	26	1					
Male	49	1.084	0.652–1.801	0.756			
**Age (years)**							
<60	34	1					
≥60	41	0.638	0.388–1.050	0.077			
**Diabetes mellitus**							
No	35	1					
Long term	25	0.906	0.439–1.872	0.790			
New onset	15	1.318	0.767–2.267	0.318			
**Tumor size (cm)**							
<4.09	34	1					
≥4.09	41	0.893	0.542–1.470	0.656			
**Lymph node involvement**							
Negative	33	1					
Positive	42	2.099	1.269–3.473	0.004	1.337	0.736–2.432	0.340
**Distant metastasis**							
No	54	1					
Yes	21	4.530	2.581–7.949	<0.001	4.022	2.074–7.798	<0.001
**IGFBP2 (ng/mL)**							
<333.9	26	1					
≥333.9	49	2.318	1.344–3.998	0.003	2.353	1.344–4.117	0.003
**CA 19-9 (U/mL)**							
<37	20	1					
≥37	55	1.160	0.650–2.069	0.616			

*Survival data were available for 75 patients; 8 were alive at the time of data analysis.

## 4 Discussion

Our study demonstrated that the plasma IGFBP2 level was significantly higher in patients with PDAC than in patients with ChPT or healthy controls in a Chinese cohort, which is consistent with a recent single-center report from the United States [10]. While IGFBP2 alone did not outperform CA19-9 in diagnostic power, as determined by sensitivity and specificity, IGFBP2 accurately identified more than one third of cases (9 out of 24) misidentified by CA19-9 in our cohort.

Rather than replacing CA 19-9 as a biomarker for PDAC, IGFBP2 may be an ideal complementary marker. The performance of CA19-9 in discriminating PDAC patients from healthy subjects is variable in the clinic, with some studies reporting barely acceptable AUC values of 0.83–0.84 [11]. In this study, we observed a very strong performance from CA19-9 in differentiating patients with PDAC from healthy controls (AUC = 0.893). The combination of IGFBP2 and CA19-9 was even stronger (AUC = 0.921). These data indicate the clinical potential of combining IGFBP2 and CA19-9 in the diagnosis of PDAC. Considering that PDAC is a heterogeneous tumor, it is reasonable to combine markers together rather than using a single one for diagnosis.

Chronic pancreatitis, especially autoimmune pancreatitis, is a risk factor for PDAC and sometimes is misdiagnosed as a malignant lesion. However, the treatments for pancreatitis and pancreatic cancer are absolutely different. Our findings show that plasma IGFBP2 was elevated exclusively in PDAC and not in ChPT or healthy controls, which is consistent with the findings of a previous study in tissue samples [12]. From the ROC curves, we also demonstrated that IGFBP2 (AUC = 0.817) was a better marker than CA19-9 (AUC = 0.779) in discrimination of PDAC from ChPT. The combination of IGFBP2 and CA19-9 even more powerfully discriminated PDAC from ChPT (AUC = 0.918).

The relationship between diabetes mellitus and PDAC has long been recognized. Most cases of pancreatic cancer-associated diabetes are of new onset, defined as onset 2 years or less preceding the diagnosis of cancer [13]. The ability to differentiate between pancreatic cancer-associated diabetes from the more common type 2 diabetes by a serological biomarker may be a great strategy for screening people at high risk of having asymptomatic pancreatic cancer.

In our analysis, the expression of IGFBP2 differed between patients with and without diabetes [14]. As far as we know, our study is the first to compare the level of IGFBP2 in PDAC patients stratified by their diabetes status. In our cohort, PDAC patients with a long history of diabetes [13] exhibited lower plasma IGFBP2 levels than those without diabetes history. This result is not surprising, because previous studies have shown that IGFBP2 has an anti-diabetes effect [15] and is expressed at lower levels in patients with diabetes than in people without diabetes [16]. Interestingly, we observed a remarkable aberrantly higher level of IGFBP2 in PDAC patients with new-onset diabetes than in PDAC patients with long-term diabetes or no diabetes. One possible explanation is that pancreatic cancer cells may secrete more IGFBP2 that cannot regulate the cancer-induced hyperglycemia. The data suggests that IGFBP2 has the potential to distinguish between the two types of diabetes in PDAC. However, one shortcoming of our study is the lack of diabetes status for the healthy controls; with that information, we could have determined the potential of IGFBP2 as an effective biomarker for PDAC screening in the diabetes risk group.

Our findings indicate that plasma IGFBP2 was elevated to the greatest extent in PDAC patients with either lymph node involvement or distant metastasis and that this elevation was associated with shorter overall survival of these patients that supports similar findings in other cancers, such as ovarian cancer [17], colorectal cancer [18], and prostate cancer [19]. Univariate and multivariate survival analyses results suggest that plasma IGFBP2 level is an independent prognostic factor when adjusted for diabetes status, lymph nodes involvement, and distant metastasis.

It is interesting that our view on the role of IGFBP2 as a predictor of patient survival in PDAC differs from that of Kendrick et al. [10]. One possible explanation for the divergence is the heterogeneity of the cohorts. Our univariate analysis considered absolutely different factors than theirs, except for IGFBP2, as predictors of survival. After adjustment by those different factors, it is possible to reach the opposite conclusion on IGFBP2’s utility in predicting survival. Different ethnic groups may be another reason for the disagreement between our conclusion and that of Kendrick et al.

Taken together, our findings suggest that the plasma IGFBP2 level is significantly elevated in PDAC and that this elevation and its extent are related to tumor progression, glucose metabolism, and prognosis. Further evaluation of IGFBP2 as a biomarker in PDAC will require larger patient cohorts with a greater variation in disease stage. Specifically, more patients with early stage PDAC (i.e., PanIn) should be included to determine the early diagnostic value of IGFBP2. Immunohistochemical data could be adopted to investigate the correlation between systemic and localized IGFBP2 levels. More mechanistic studies are imperative to show how IGFBP2 is involved in these referred processes in PDAC. The integrin/ILK/NF-κB [20], IGF1R/PI3K/AKT [21], and Wnt/β-catenin [22] pathways, as well as certain micro-RNAs have been proven to regulate or be regulated by IGFBP2 in a variety of malignant tumors. Characterization of IGFBP2-mediated pathways in pancreatic cancer cells may provide molecular insights into the progression of pancreatic cancer and suggest potential therapeutic targets for pancreatic cancer patients with elevated IGFBP2. In conclusions, this randomized, retrospective study has demonstrated that plasma IGFBP2 is significantly elevated in Chinese PDAC patients compared to ChPT or healthy controls. IGFBP2 is a complementary biomarker for PDAC because the combination of it with CA19-9 is more accurate in diagnosis than using CA19-9 alone. Higher plasma IGFBP2 is correlated with the new onset diabetes, lymph node involvement, distant metastasis, and advanced stage. Our findings also assert that elevated plasma IGFBP2 is an independent prognostic marker for PDAC. To further illustrate the clinic value of IGFBP2 in PDAC, a larger cohort with more early-stage patients and greater clinicopathological variation is necessary for future study.

## References

[1] Sung H, Ferlay J, Siegel RL, Laversanne M, Soerjomataram I, Jemal A, et al. Global Cancer Statistics 2020: GLOBOCAN estimates of incidence and mortality worldwide for 36 cancers in 185 countries. CA Cancer J Clin. 2021;71(3):209–49. 10.3322/caac.21660. 33538338

[2] Siegel RL, Miller KD, Wagle NS, Jemal A. Cancer statistics, 2023. CA Cancer J Clin. 2023;73(1):17–48. 10.3322/caac.21763. 36633525

[3] Chen J, Wang H, Zhou L, Liu Z, Tan X. A combination of circulating tumor cells and CA199 improves the diagnosis of pancreatic cancer. J Clin Lab Anal. 2022;36(5):e24341. 10.1002/jcla.24341. PMC9102772 35334495

[4] Baxter RC. IGF binding proteins in cancer: mechanistic and clinical insights. Nat Rev Cancer. 2014;14(5):329–41. 10.1038/nrc3720. 24722429

[5] Wei LF, Weng XF, Huang XC, Peng YH, Guo HP, Xu YW. IGFBP2 in cancer: pathological role and clinical significance (Review). Oncol Rep. 2021;45(2):427–38. 10.3892/or.2020.7892. 33416173

[6] Prayudi PKA, Budiana ING, Mahayasa PD, Surya I, Wiradnyana A, Suwiyoga K. Diagnostic accuracy of serum insulin-like growth factor-binding protein 2 for ovarian cancer. Int J Gynecol Cancer. 2020;30(11):1762–7. 10.1136/ijgc-2020-001479. 32817171

[7] Resovi A, Bani MR, Porcu L, Anastasia A, Minoli L, Allavena P, et al. Soluble stroma-related biomarkers of pancreatic cancer. EMBO Mol Med. 2018 Aug;10(8):e8741. 10.15252/emmm.201708741. PMC6079536 29941541

[8] Wlodarczyk B, Borkowska A, Wlodarczyk P, Malecka-Panas E, Gasiorowska A. Serum levels of insulin-like growth factor 1 and insulin-like growth factor-binding protein 2 as a novel biomarker in the detection of pancreatic adenocarcinoma. J Clin Gastroenterol. 2020;54(9):e83–8. 10.1097/MCG.0000000000001297. 31851103

[9] McCaffery I, Tudor Y, Deng H, Tang R, Suzuki S, Badola S, et al. Putative predictive biomarkers of survival in patients with metastatic pancreatic adenocarcinoma treated with gemcitabine and ganitumab, an IGF1R inhibitor. Clin Cancer Res. 2013;19(15):4282–9. 10.1158/1078-0432.CCR-12-1840. 23741071

[10] Kendrick ZW, Firpo MA, Repko RC, Scaife CL, Adler DG, Boucher KM, et al. Serum IGFBP2 and MSLN as diagnostic and prognostic biomarkers for pancreatic cancer. HPB (Oxford). 2014;16(7):670–6. 10.1111/hpb.12199. PMC4105906 24308545

[11] Brand RE, Nolen BM, Zeh HJ, Allen PJ, Eloubeidi MA, Goldberg M, et al. Serum biomarker panels for the detection of pancreatic cancer. Clin Cancer Res. 2011;17(4):805–16. 10.1158/1078-0432.CCR-10-0248. PMC3075824 21325298

[12] Saraswat M, Joenvaara S, Seppanen H, Mustonen H, Haglund C, Renkonen R. Comparative proteomic profiling of the serum differentiates pancreatic cancer from chronic pancreatitis. Cancer Med. 2017;6(7):1738–51. 10.1002/cam4.1107. PMC5504330 28573829

[13] Pereira SP, Oldfield L, Ney A, Hart PA, Keane MG, Pandol SJ, et al. Early detection of pancreatic cancer. Lancet Gastroenterol Hepatol. 2020;5(7):698–710. 10.1016/S2468-1253(19)30416-9. PMC7380506 32135127

[14] Huang H, Dong X, Kang MX, Xu B, Chen Y, Zhang B, et al. Novel blood biomarkers of pancreatic cancer-associated diabetes mellitus identified by peripheral blood-based gene expression profiles. Am J Gastroenterol. 2010;105(7):1661–9. 10.1038/ajg.2010.32. 20571492

[15] Hedbacker K, Birsoy K, Wysocki RW, Asilmaz E, Ahima RS, Farooqi IS, et al. Antidiabetic effects of IGFBP2, a leptin-regulated gene. Cell Metab. 2010;11(1):11–22. 10.1016/j.cmet.2009.11.007. 20074524

[16] Heald AH, Kaushal K, Siddals KW, Rudenski AS, Anderson SG, Gibson JM. Insulin-like growth factor binding protein-2 (IGFBP-2) is a marker for the metabolic syndrome. Exp Clin Endocrinol Diabetes. 2006;114(7):371–6. 10.1055/s-2006-924320. 16915540

[17] Periyasamy A, Gopisetty G, Subramanium MJ, Velusamy S, Rajkumar T. Identification and validation of differential plasma proteins levels in epithelial ovarian cancer. J Proteom. 2020;226:103893. 10.1016/j.jprot.2020.103893. 32634479

[18] Bhardwaj M, Terzer T, Schrotz-King P, Brenner H. Comparison of proteomic technologies for blood-based detection of colorectal cancer. Int J Mol Sci. 2021;22(3):1189. 10.3390/ijms22031189. PMC7865621 33530402

[19] Watts EL, Perez-Cornago A, Fensom GK, Smith-Byrne K, Noor U, Andrews CD, et al. Circulating insulin-like growth factors and risks of overall, aggressive and early-onset prostate cancer: a collaborative analysis of 20 prospective studies and Mendelian randomization analysis. Int J Epidemiol. 2023;52(1):71–86. 10.1093/ije/dyac124. PMC9908067 35726641

[20] Gao S, Sun Y, Zhang X, Hu L, Liu Y, Chua CY, et al. IGFBP2 activates the NF-kappaB pathway to drive epithelial–mesenchymal transition and invasive character in pancreatic ductal adenocarcinoma. Cancer Res. 2016;76(22):6543–54. 10.1158/0008-5472.CAN-16-0438. PMC5315491 27659045

[21] Liu Y, Nelson MV, Bailey C, Zhang P, Zheng P, Dome JS, et al. Targeting the HIF-1alpha-IGFBP2 axis therapeutically reduces IGF1-AKT signaling and blocks the growth and metastasis of relapsed anaplastic Wilms tumor. Oncogene. 2021;40(29):4809–19. 10.1038/s41388-021-01907-1. PMC8319145 34155347

[22] Verma BK, Kondaiah P. Regulation of beta-catenin by IGFBP2 and its cytoplasmic actions in glioma. J Neurooncol. 2020;149(2):209–17. 10.1007/s11060-020-03596-4. 32803659

